# Initial pH and mortality in patients with exacerbations of COPD and pneumonia treated with NIV in a teaching hospital critical care unit

**DOI:** 10.1186/cc14333

**Published:** 2015-03-16

**Authors:** IM Goodhart, MC Faulds, S Lobaz, AJ Glossop

**Affiliations:** 1 Sheffield Teaching Hospitals NHS FT, Sheffield, UK

## Introduction

Bilevel non-invasive ventilation (NIV) is an established therapy in chronic obstructive pulmonary disease (COPD) but conflicting evidence exists for its use in patients with pneumonia. Initial arterial pH <7.25 is used as a marker of severity and need for admission to critical care (CC) [1]. We examined the impact of pH and condition on outcome in patients with acute respiratory failure (ARF) of mixed aetiology treated with NIV.

## Methods

Data were collected retrospectively for a 5-year period from 2008 to 2013 using the Metavision electronic patient record. We identified all patients admitted with ARF treated with bilevel NIV. Patients who received continuous positive airway pressure or had a primary surgical problem were excluded. We recorded primary cause of respiratory failure, arterial blood gas values and mortality.

## Results

A total of 145 patients were identified. Mean age was 64 and 51% were male. The primary diagnosis was pneumonia in 69 patients and exacerbation of COPD in 57. The overall mortality was 19% on CC and 39% at 1 year. In patients with COPD, infective exacerbations had a higher CC mortality (17%) compared with non-infective (0%). However, by 1 year the mortality was 28% in infective and 29% in non-infective. Patients with pneumonia had a higher mortality on CC (25%) and at 1 year (48%). Patients with an initial pH <7.25 were less likely to survive. The mortality at discharge from CC was 16% (pH ≥7.25) and 26% (pH <7.25) but narrowed to 38% and 39% by 1 year. When subdivided, it was found that patients with infective COPD and pH <7.25 had the lowest 1-year mortality (17%) while those with pneumonia and pH <7.25 had the highest mortality (67%).

## Conclusion

NIV is used in our unit with comparable success rates to published series [2,3]. COPD patients responded well to NIV, while patients with pneumonia treated with NIV have the highest mortality. A low presenting pH is associated with a higher mortality in patients with pneumonia treated with NIV. However, in COPD patients, pH <7.25 is not associated with higher mortality in CC or at 1 year. Further work defining the precise role of pH as a prognostic indicator is warranted.
